# Increased N-Glycosylation Efficiency by Generation of an Aromatic Sequon on N135 of Antithrombin

**DOI:** 10.1371/journal.pone.0114454

**Published:** 2014-12-08

**Authors:** Sonia Águila, Irene Martínez-Martínez, Gilda Dichiara, Ricardo Gutiérrez-Gallego, José Navarro-Fernández, Vicente Vicente, Javier Corral

**Affiliations:** 1 Centro Regional de Hemodonación, Hospital Morales Meseguer, Universidad de Murcia, IMIB, Murcia, Spain; 2 Division of Cardiovascular Sciences, Laboratory of Thrombosis and Haemostasis, Centre for Applied Medical Research, University of Navarra, Pamplona, Spain; 3 Bioanalysis Group, IMIM-Hospital del Mar, Department of Experimental and Health Sciences, University Pompeu Fabra (UPF), Barcelona, Spain; 4 Protein and Peptide Chemistry, Anapharm Biotech, Barcelona, Spain; Weizmann Institute of Science, Israel

## Abstract

The inefficient glycosylation of consensus sequence on N135 in antithrombin explains the two glycoforms of this key anticoagulant serpin found in plasma: α and β, with four and three N-glycans, respectively. The lack of this N-glycan increases the heparin affinity of the β-glycoform. Recent studies have demonstrated that an aromatic sequon (Phe-Y-Asn-X-Thr) in reverse β-turns enhances N-glycosylation efficiency and stability of different proteins. We evaluated the effect of the aromatic sequon in this defective glycosylation site of antithrombin, despite of being located in a loop between the helix D and the strand 2A. We analyzed the biochemical and functional features of variants generated in a recombinant cell system (HEK-EBNA). Cells transfected with wild-type plasmid (K133-Y-N135-X-S137) generated 50% of α and β-antithrombin. The S137T, as previously reported, K133F, and the double mutant (K133F/S137T) had improved glycosylation efficiency, leading to the secretion of α-antithrombin, as shown by electrophoretic and mass analysis. The presence of the aromatic sequon did not significantly affect the stability of this conformationally sensitive serpin, as revealed by thermal denaturation assay. Moreover, the aromatic sequon hindered the activation induced by heparin, in which is involved the helix D. Accordingly, K133F and particularly K133F/S137T mutants had a reduced anticoagulant activity. Our data support that aromatic sequons in a different structural context from reverse turns might also improve the efficiency of N-glycosylation.

## Introduction

The N-glycosylation is a post-translational modification, crucial for membrane and secreted proteins, which begins in the endoplasmic reticulum with a common precursor oligosaccharide, a dolichol-bound oligosaccharide (Glc_3_Man_9_GlcNAc_2_), which is conserved in eukaryotes. It links to asparagines in the context of a tripeptide consensus sequence N-X-S/T and rarely the N-X-C motif, where X may be any amino acid except proline [1], [2]. Next, the protein is transported to the Golgi apparatus where the carbohydrate elongation and specific modifications are produced, rendering variable glycan structures [3]. The N-linked glycan can be dispensable for the function of many glycoproteins but, in some cases, it may affect protein conformation or folding, and even the function and specificity of interaction [4], [5]. Moreover, glycosylation plays a key role in the transit system of proteins out of the endoplasmic reticulum, in their stability or half-life, in the sensitivity to proteases or others reactive substances, and in their immunogenicity [6], [7]. When glycosylation does not occur properly or is inhibited, proteins may form aggregates and are degraded via proteasome [8], [9]. The urgent need for glycan-defined glycoproteins in both detailed structure-function relationship studies and therapeutic applications has stimulated the development of various methods for manipulating protein glycosylation [5], [10]–[12]. In this framework, particular interest has two recent studies evaluating the effect of an aromatic residue before the N-X-T, on the glycosylation efficiency and the stability of proteins [13]–[15]. The introduction of a phenylalanine, two residues before the asparagine of N-glycosylation consensus sequence, situated in reverse turns increased the glycosylation efficacy and the stability of different proteins due to the side chain interactions of phenylalanine with the hydrophobic α face of GlcNAc1 on asparagine [13], [14]. This study also suggested that the presence of a threonine two residues after asparagine in the consensus sequence favors a more compact structure [13]. A more recent study from the same group also showed that the increased of the stability provoked by the interaction between the glycan and aromatic ring is more notable if this aromatic sequon is located in type I β-bulge turns other than reverse turns [15]. However, as far as we know no further study has evaluated the relevance of an aromatic sequon in other structural context or localization.

Antithrombin is a key anticoagulant that inhibits multiple procoagulant proteases such as thrombin and factor Xa (FXa) by an efficient suicide mechanism. The inhibitory mechanism of antithrombin, like other members of the serpin superfamily, requires a great conformational flexibility [16], [17]. Antithrombin has four potential N-glycosylation sites: 96; 135, 155, and 192, but N135 is inefficiently glycosylated. Therefore, the presence or absence of a glycan at N135 yields the two main glycoforms of antithrombin identified in plasma: α-antithrombin, which is fully glycosylated; and β-antithrombin, where the potential glycosylation site at N135 is not occupied [18], [19]. This event has been explained by the presence of a serine instead of a threonine in the consensus sequence of N135 [20], although the exact mechanism is not fully understood. The consequences of the reduced glycosylation are not restricted to physic differences [a smaller size and an increased pI in the β-glycoform (56 KDa and pI: 5.625) compared with the α-glycoform (58 KDa and pI: 5.375)], as it also has functional effect on the activation by heparin or pentasaccharide (the essential oligosaccharide sequence for antithrombin activation) (S1 Figure), which is relevant for the anticoagulant role of this serpin. Native antithrombin presents the reactive center loop partially inserted. The binding of heparin triggers conformational changes that lead to the full expulsion of the reactive center loop, increasing the reactivity (activated antithrombin) [21], [22]. Thus, the close localization of this N-glycan to the heparin binding site explains why β-antithrombin has higher heparin affinity than the counterpart (α-antithrombin) [18], [23]. Additionally, the lack of glycan in β-glycoform has also been involved in its faster clearance rate [24].

The aim of this study was to evaluate the effect of an aromatic sequon (F-Y-N-X-T/S) on the glycosylation consensus sequence of N135 of antithrombin. To achieve this aim, we analyzed the glycosylation efficiency, inhibitory function, heparin affinity and structural stability of different antithrombin mutants. This is the first study evaluating the effect of this aromatic sequon in a different structural context, since the N135-K136-S137 glycosylation consensus sequence of antithrombin lays in a loop between a α-helix and a β-strand, but not in a reverse turn, as it has been described previously.

## Materials and Methods

### Site directed mutagenesis and recombinant expression

We used the pCEP4-AT plasmid containing the cDNA sequence of human antithrombin and Human Embryonic Kidney cells expressing the Epstein Barr Nuclear Antigen 1 (HEK-EBNA) cells, generously provided by Prof. J. Huntington (CIMR, Cambridge, UK). This plasmid, commonly used to produce recombinant antithrombin in HEK-EBNA cells, had the S137A mutation to only produce β-antithrombin [20]. Firstly, we returned to the wild type (WT) sequence (S137), and then we generated the following mutations, K133F, S137T and K133F/S137T by site directed mutagenesis using the Stratagene Quik Change Site-Directed Mutagenesis kit (Agilent, Madrid, Spain) and the appropriate primers (S1 Table). HEK-EBNA cells were grown to 80% confluence at 37°C, 5% CO_2_, in DMEM with GlutaMAX-I medium (Invitrogen, Barcelona, Spain) supplemented with 5% fetal bovine serum (Sigma-Aldrich, Madrid, Spain). Transfection was performed by addition of plasmid (200 µg/mL) that it was preincubated for 30 minutes in serum-free OptiMEM culture medium with Lipofectamine LTX reagent (Invitrogen, Barcelona, Spain) according to the manufacturer's protocol. Sixteen hours post-transfection, cells were washed with PBS and exchanged into CD-CHO medium (Invitrogen, Barcelona, Spain) supplemented with 4 mM L-glutamine and 0.25 mg/mL Geneticin (Invitrogen, Barcelona, Spain). Cells were grown to confluence and media was harvested every 42 h for 11 days.

Cells were extensively washed with sterile PBS and then lysated with 50 µl of lysis buffer (10 mM TrisHCl, 0.5 mM DTT, 0.035% SDS, 1mM EGTA, 50 mM sodium fluoride, 50 µM sodium orthovanadate, 5 mM benzamidine and 20 mM phenylmethylsulphonyl fluoride). The homogenate was centrifuged at 12000 rpm for 10 minutes at 4°C and supernatants were kept stored at −80°C. Separation of proteins was evaluated by SDS-PAGE under reducing conditions and immunoblotting. Monoclonal anti β-actin (Sigma-Aldrich, Madrid, Spain) was used to check sample loading.

### Protein purification and electrophoretic evaluation

Recombinant proteins were purified by heparin affinity chromatography on 5 mL HiTrap Heparin columns (GE Healthcare, Barcelona, Spain), using an ÄKTA Purifier (GE Healthcare, Barcelona, Spain) in 50 mM Tris-HCl, pH 7.4 buffer where a gradient from 0 to 3 M NaCl was applied to elute the proteins. Next, fractions containing antithrombin were further purified by anion exchange chromatography on 1 mL HiTrap Q columns (GE Healthcare, Barcelona, Spain) by applying a gradient from 0 to 1 M NaCl in 50 mM Tris-HCl pH 7.4. Those fractions containing pure antithrombin were finally desalted and stored at −70°C. Purity of proteins was evaluated by 8% SDS-PAGE by silver staining, as indicated elsewhere [25].

### MALDI-TOF-MS analysis

A solution of 3,5-dimethoxy-4-hydroxycinnamic acid (10 g/L) in acetonitrile (ACN)/water/trifluoroacetic acid (TFA) (50:50:0.1 by vol.) was chosen as matrix for protein analysis. Experiments were carried out on a 4800 Plus MALDI TOF/TOF Analyzer (ABSciex), equipped diode-pumped, solid-state laser. Recorded data were processed with Data Explorer Software (Applied Biosystems, California, USA).

### Determination of denaturing temperature

Thermal denaturation of antithrombin variants was monitored by changes in tryptophan fluorescence, with excitation wavelength at 280 nm and emission wavelength at 345 nm using the temperature controlled Agilent Caryeclipse fluorometer (Agilent, Madrid, Spain). A 0.5°C/min rate of temperature change was used. Antithrombin concentration was 300 nM in phosphate buffer pH 7.4 and *I* = 0.15. The data was fitted to the Van't Hoff equation:
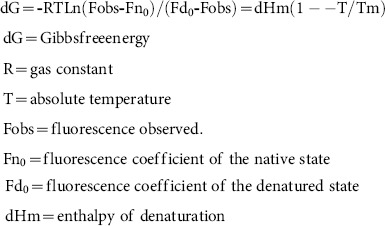



Fluorescence coefficients were determined from the linear parts of plots.

### Determination of dissociation equilibrium constant (K_D_) and inhibitory function

Equilibrium dissociation constant for the antithrombin-heparin interaction was determined by intrinsic fluorescence analysis performed essentially as described previously [26], [27]. Briefly, changes in intrinsic fluorescence of antithrombin (25 nM) upon titration of unfractionated heparin (Rovi, Madrid, Spain) were monitored at 340 nm on a Hitachi F4500 spectrofluorometer, with excitation at 280 nm and using bandwidths of 3.5 nm for both excitation and emission. All titrations were carried out at room temperature under physiological ionic strength (*I* = 0.15) in 20 mM NaPO_4_, 100 mM NaCl, 0.1 mM EDTA, 0.1% polyethylene glycol 8000, pH 7.4. Fluorescence emission intensity was taken as the average of 100 measurements recorded at one second intervals for each addition of heparin. Data were fitted as previously described [26], [27].

Anti-FXa activity was evaluated using 400 ng of total purified protein and the chromogenic method, as described elsewhere [28].

### Stopped flow

The kinetics of heparin binding were monitored continuously from the increase in protein fluorescence in an spectrometer π*-180 (Applied Photophysics) connected to a stopped-flow kinetic sample handling unit, as described previously [29] with a range of concentration of pentasaccharide from 4 nM to 30 µM (always in at least 5-fold molar excess of pentasaccharide to ensure under pseudo-first order conditions). For reactions at inhibitor concentrations less than 1µM, two reaction curves were typically averaged for each rate constant determination. All measurements were conducted at 25°C in pH 7.4 buffer consisting of 20 mM sodium phosphate; 0.1 mM EDTA; 0.1% polyethylene glycol 8000; and 0.1 M NaCl to achieve ionic strengths of 0.15.

### Statistical analysis

For the intrinsic fluorescence and anti-FXa activity assays, differences between mutants were analyzed by the Student *t*-test with two-tailed *p*-value. A significance level (*p*) of 0.05 was applied in all the statistical analyses, which were performed using the Prism5 software package (GraphPad software, San Diego, CA, USA).

## Results

### Evaluation of the glycosylation efficiency

**Figure 1:**
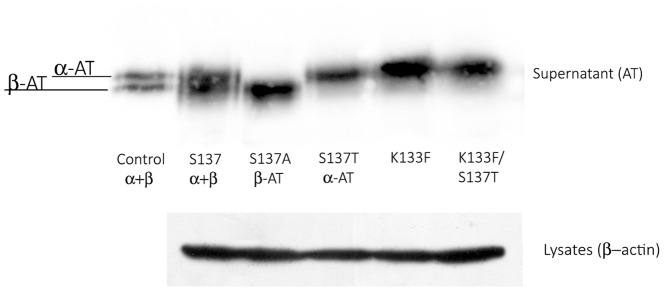
Expression of antithrombin variants. SDS-PAGE under reducing conditions and western blot of recombinant antithrombin variants from intracellular lysates or conditioned medium of HEK-EBNA cells 24 h after transfection. As loading control in cellular lysates we showed the expression of β-actin. As control of electrophoretic mobility we used a mixture of α and β-antithrombin purified from plasma of healthy subjects.

**Figure 2:**
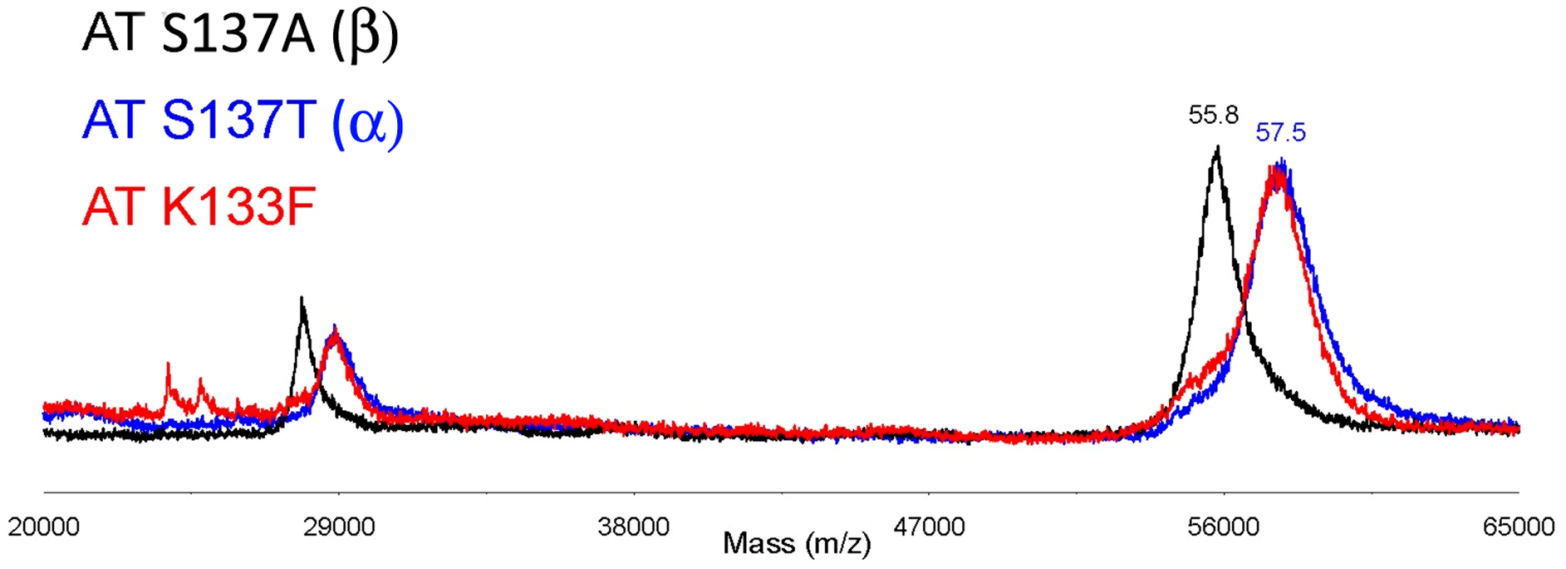
MALDI-TOF mass spectra of purified antithrombin variants.

Firstly, all variants rendered similar amounts of antithrombin to the conditioned medium 24 h after transfection (Fig. 1). These results support that no one significantly impaired the intracellular traffic and secretion of variants. Then, we compared the efficiency of glycosylation at Ans135 of the three different mutations affecting the WT glycosylation consensus sequence (**N135**-K136-**S137**) by evaluating the electrophoretic mobility of the secreted protein to the medium. As shown in Fig. 1, the transfection with the WT plasmid resulted in a similar production of α and β-glycoforms. Moreover, the mutation of S137 residue to Ala (S137A) only produced β-antithrombin [20] (Fig. 1). In contrast, the mutation of this residue to threonine (S137T) turned into the production of α-antithrombin (Fig. 1), as previously described [20]. Interestingly, the inclusion of an aromatic residue by the K133F mutation, two residues before N135 in a WT context (**K133**-A134-**N135**-K136-**S137**), enhanced sequon occupancy to ∼100% (Fig. 1). Finally, the combination of both mutations, K133F and S137T, also produced only α-antithrombin (Fig. 1). These results were confirmed by mass analysis of purified proteins (MALDI-TOF MS) (Fig. 2).

### Thermodynamic stability of purified antithrombin variants

After purification, proteins were concentrated and used for denaturation analysis by following the intrinsic fluorescence change. A moderate increased denaturation temperature (1.1°C) was observed for the K133F mutant compared with control (S137T) (Table 1), although the double mutant (K133F/S137T) had the same melting temperature than the variant without aromatic sequon (S137T) (Table 1).

**Table 1:** Melting temperatures of antithrombin variants determined by intrinsic fluorescence changes, as described in Materials and Methods section.

Variant	*Glycans (n°)*	*Tm* (X ± SD)
S137A	3	56.2±0.2
S137T	4	57.6±0.0
K133F/S137A	3	57.3±0.1
K133F	4	58.7±0.0
K133F/S137T	4	57.3±0.2

Each value was calculated as the mean (X) ± standard deviation (SD) of two independent measurements.

### Heparin affinity and functionality

The effect of these mutations was also evaluated on two functional features of antithrombin: heparin affinity and inhibition of FXa.

We analyzed the influence of the aromatic sequon on the heparin affinity determined by the changes of the intrinsic fluorescence caused by the titration with unfractionated heparin. As shown in table 2, the β-glycoform (S137A) had higher heparin affinity than the α-glycoform (S137T), as previously reported [30]. The aromatic sequon without glycan (K133F/S137A; three glycans) increased the dissociation constant (*K_D_*) 1.5-fold with respect to control, S137A (Table 2). Moreover, the glycosylation of this aromatic sequon (K133F; four glycans) presented a greater increase of *K_D_* (∼2-fold) compared to the control (S137T) (Table 2). Finally, the double mutant (K133F/S137T) had more affected the heparin affinity than control (S137T), a 4-fold lower *K_D_* (Table 2). Therefore, the phenylalanine together with the glycan reduced the heparin affinity. This aromatic residue is not by itself the responsible for this effect.

**Table 2:** Equilibrium dissociation constants for the binding of unfractionated heparin by antithrombin variants.

Variant	*Glycans (n^o^)*	*K_D_* (nM)	ΔF max/Fo ×100
S137A	3	37.6±3.1	41.0
S137T	4	48.9±2.5	43.4
K133F/S137A	3	55.5±4.9	42.0
K133F	4	81.4±3.6	44.1
K133F/S137T	4	195.4±4.0	44.5

Dissociation constants (*K_D_*) and maximal fluorescence increase were measured by global fitting of fluorescence titrations with unfractionated heparin as described in Materials and Methods section. The differences between mutants were tested for statistical significance by paired *t*-test, where p-values were <0.005.

**Figure 3:**
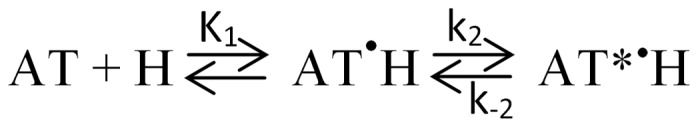
Function of antithrombin variants. Anti-FXa activity of antithrombin proteins secreted to the conditioned medium in presence of heparin. Results are expressed as a percentage of the activity of the S137T variant. Each bar represents the mean ± standard deviation (SD) of two independent experiments performed in duplicate. The differences between mutants were tested by paired *t*-test (p-value). The “*” indicated differences statistically significant with p<0.05.

Functional analysis of FXa inhibition by using chromogenic assays verified the consequences of the impaired heparin affinity induced by the aromatic sequon. Thus, the β-glycoform generated by mutation at residue 137 (S137A) had similar inhibitory activity (anti-FXa) than the α-glycoform used as reference (S137T), as it has been described previously [31] (Fig. 3). However, if the aromatic sequon is glycosylated, the variant had reduced anti-FXa activity, whose reduction is particularly severe for the double mutant (80.3% and 55.2% K133F and K133F/S137T, respectively) (Fig. 3).

### Kinetics of Heparin Binding

**Figure 4:**
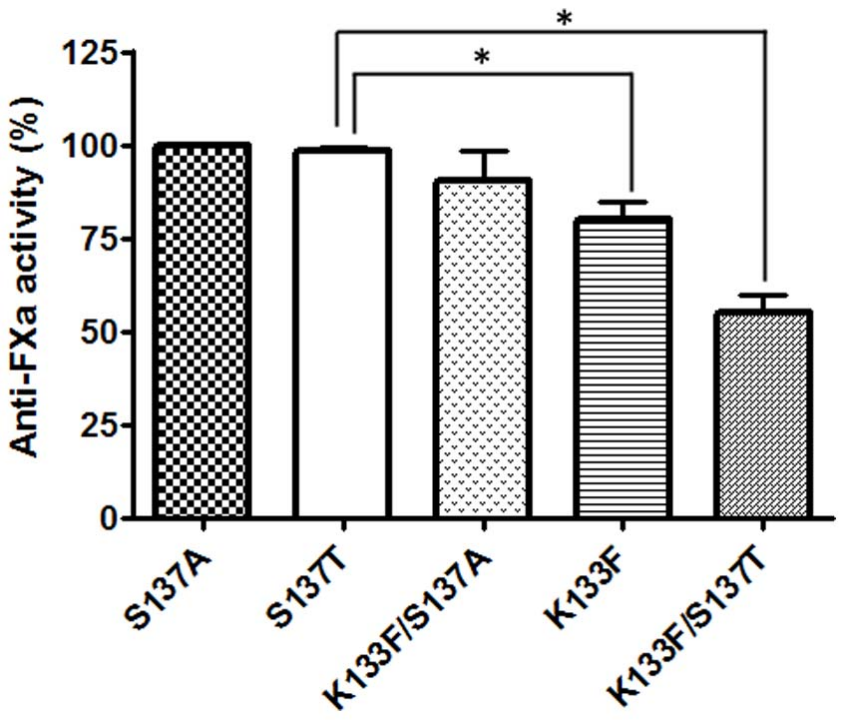
Scheme of binding of antithrombin and heparin. Initial rapid equilibrium, **K_1_**, between antithrombin, **AT**, and pentasaccharide, **H**, leads to complex, **AT.H**, followed by rapid conformational change via **k_2_** to a high heparin affinity, highly fluorescence complex, AT*.H.

The pentasaccharide binds to antithrombin in an initial rapid equilibrium step characterized by a dissociation constant, K_1_, and then induces a conformational changes in the protein with forward and reverse rate constants, k_+2_ and k_−2_ (Fig. 4). The binding of antithrombin variants with pentasaccharide were monitored by the increase of intrinsic fluorescence of protein under pseudo-first order conditions. The aromatic ring of phenylalanine is not the responsible for this effect by itself, since only moderately affected K_1_ and k_2_ compared to β-antithrombin (S137T) (Table 3). However, the presence of aromatic residue (K133F or K133F/S137T mutants) together with the presence of glycan dramatically affected the allosteric activation by pentasaccharide, as K_1_; k_+2_ and k_−2_ were not measurable in the same conditions as controls (Table 3). Moreover, the presence of an additional glycan on α-antithrombin (S137T) had also weak impact on the kinetic heparin binding constants of the pentasaccharide (Table 3).

**Table 3:** Stopped-flow pentasaccharide binding data were measured as described in Materials and Methods section.

Variant	*Glycans (n^o^)*	K_1_ (µM)	K_2_ (s^−1^)	K_−2_ (s^−1^)
S137A	3	5.0±0.8	374.0±5.4	0.6±0.2
S137T	4	20.2±0.6	338±2.3	2.2±0.6
K133F/S137A	3	26.5±0.5	461.0±1.8	0.7±0.4
K133F	4	_	_	_
K133F/S137T	4	_	_	_

## Discussion

**Figure 5:**
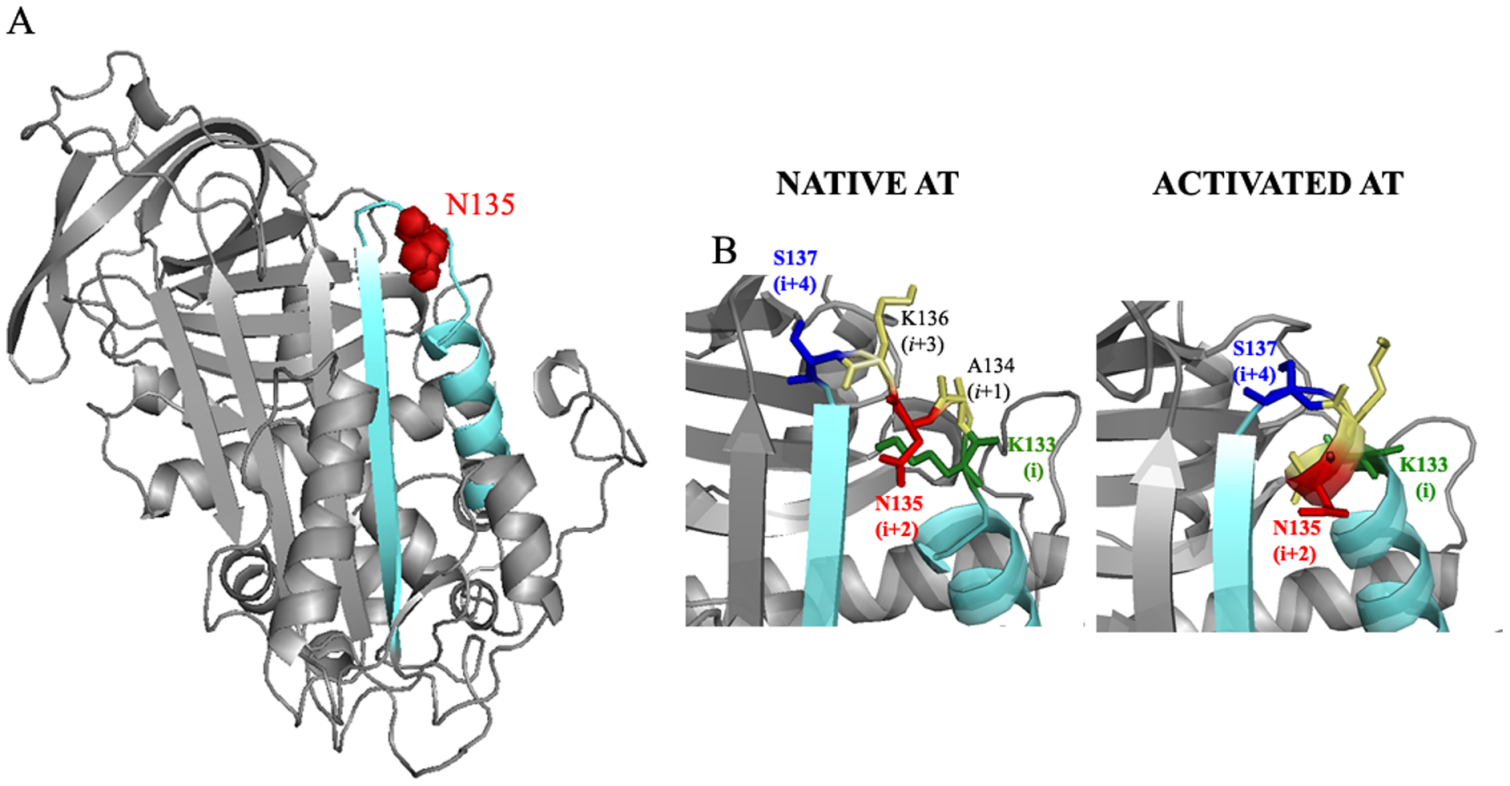
Ribbon diagram of antithrombin. A) Structural representation of the localization of N135 (red spheres) in native antithrombin (PDB code 1T1FA). Strand 2A and helix D are displayed in cyan. B) Stick representation of the WT glycosylation consensus sequence (K133/S137 or (*i*) and (*i*+4) position) on native and activated antithrombin (AT) (PDB code 1T1F and 2GD4, respectively). All structures were rendered in Pymol (www.pymol.org).

Protein glycosylation is a complex post-translational modification crucial for proteins, since it determines functional specificity, clearance and stability. These features together with the increasing use of recombinant proteins, particularly for therapeutic applications, highlight the relevance of emerging glycoengineered technologies aiming to reduce heterogeneity of glycoforms, to improve protein stability or increase its half life [7], [11], [12], [14], [32]. The final goal is to achieve the desired therapeutic efficacy of the recombinant molecule. In this framework, two recent studies from the same group have shown that the N-linked glycans have an influence on the folding and enhance the stability of proteins [13]–[15]. These works show that the incorporation of an aromatic sequon (Phe–Y–Asn–X–Thr, where Y can likely be any amino acid, and X is any amino acid but proline) is an interesting strategy for enhancing an efficient glycosylation and for increasing the stability of many proteins that harbor reverse β-turns [13]–[15]. Moreover, the statistical analysis of 506 crystallographic structures of proteins suggests that the presence of aromatic residues before an asparagine in occupied N-glycosylation sites is a significantly above average occurrence [33]. Our study demonstrated that the introduction of aromatic sequon may also be useful to improve the glycosylation efficiency of antithrombin in other structural contexts. The WT antithrombin, with N135-K136-S137 glycosylation consensus sequence, is inefficiently glycosylated, as the 50% of molecules are glycosylated at this position. Although this sequence is not located in the reverse turn (none of the glycosylation sites of antithrombin is placed in a reverse β-turn), but in a loop between helix D and strand 2A (Fig. 5), the presence of an aromatic residue (Phe) two residues before the asparagine residue (*i* and *i*+*2*, respectively, using the nomenclature of Culyba et al., 2011) allows a complete N-glycosylation of this site, similarly to that caused by the S137T mutation [20]. Interestingly, no antithrombin across species has aromatic aminoacids (Phe, Tyr or Trp) one or two residues before the homologous N135 (S2 Figure).

The aromatic sequon in reverse β-turns also increased stability of different proteins (rat CD2 adhesion domain, human muscle acyl phosphatase and WW domain of the human protein Pin1) [13]. This effect was explained by the hydrophobic interactions between the α-face of GlcNAc1 and the aromatic ring of phenylalanine at (*i*) position together with the C-H/π interaction [13], [15]. Thus, we evaluated the effect of the aromatic sequon on the structural stability of this sensitive serpin. The thermal denaturation study of purified variants confirmed that the presence of a phenylalanine at (*i*) position, despite improving the efficiency of N-glycosylation on consensus sequence of N135, does not significantly increase the stability of this protein. It is possible that the position of the aromatic sequon in a loop might explain this effect, but we think that the stability caused by the aromatic sequon cannot compensate the instability of the stressed native conformation of antithrombin, i.e. a metastable conformation required for an efficient inhibitory mechanism [34], [35] that also provides antithrombin vulnerable to environmental or genetic factors, like other serpins [36]–[38].

Moreover, the aromatic sequon might have other structural consequences on antithrombin, since this anticoagulant protein requires the activation induced by heparin to achieve a full anticoagulant activity (activated conformation) [21], [39] and this glycosylation site (N135) is located close to the heparin binding site (Fig. 5). The heparin affinity analysis, the stopped flow assay and the functional studies revealed that the aromatic sequon together with the carbohydrate might hamper the conformational changes triggered by heparin activation [40]. It is possible that the side chain interactions of aromatic ring with the hydrophobic α face of GlcNAc1 described by Culyba et al., 2011 [13] that do not provide enough strength to improve the stability of this serpin could fortify the native conformation. Further studies are required to verify this hypothesis.

In conclusion, the presence of an aromatic sequon generated by molecular engineering on the defective glycosylation consensus sequence of human antithrombin allows an efficient N-glycosylation process, leading to a homogeneous production of recombinant α-antithrombin, despite this sequence is not located in a reverse β-turn. This modification does not significantly increases the stability of antithrombin, but the presence of phenylalanine together with the carbohydrate hampered the activation induced by heparin and therefore, it affects its function. Further studies are required to verify whether or not this strategy might be useful to improve glycosylation efficiency and stabilization of proteins in other tertiary structures, apart from reverse turns, which may be useful in glycoengineering [12], [32], [41].

## Supporting Information

S1 Figure **Structure of pentasaccharide.** The structure of the essential pentasaccharide sequence present in heparin that is able to activate antithrombin [42].
(TIF)

S2 Figure **Aligned sequences of 26 antithrombins of different species.** These sequences are available at Uniprot (www.uniprot.org/uniprot/). Alignments were performed by clustalW2 (www.ebi.ac.uk/Tools/msa/clustalw2/). The N-glycosylation sequence is underlined and the lysine residue bolded. We excluded fish and other organism sequences because they have not glycosylation consensus sequence on this position.
(TIFF)

S1 Table **Primers sequence used for site directed mutagenesis.**
(PDF)
