# Correction: Increased N-Glycosylation Efficiency by Generation of an Aromatic Sequon on N135 of Antithrombin

**DOI:** 10.1371/journal.pone.0122177

**Published:** 2015-03-23

**Authors:** The PLOS ONE Staff

The images for Figs. 3 and 4 are incorrectly switched. The image that appears as Fig. 3 should be Fig. 4, and the image that appears as Fig. 4 should be Fig. 3. The figure legends appear in the correct order.

**Fig 3:**
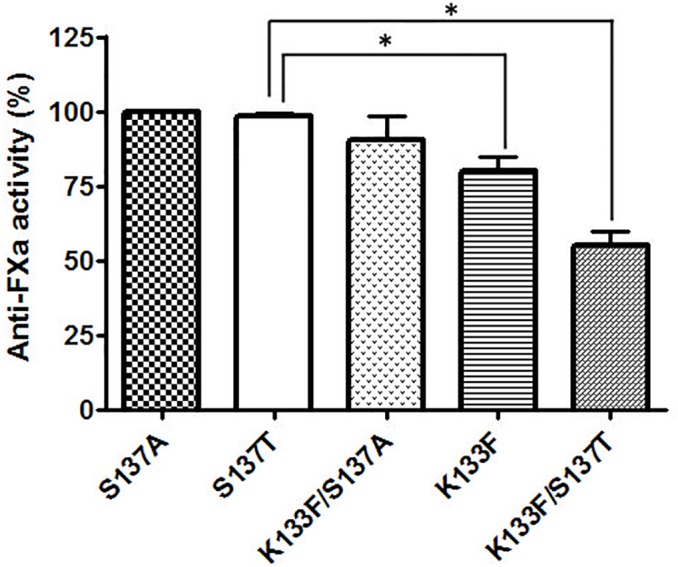
Function of antithrombin variants. Anti-FXa activity of antithrombin proteins secreted to the conditioned medium in presence of heparin. Results are expressed as a percentage of the activity of the S137T variant. Each bar represents the mean ± standard deviation (SD) of two independent experiments performed in duplicate. The differences between mutants were tested by paired *t*-test (p-value). The “*” indicated differences statistically significant with p<0.05.

**Fig 4:**
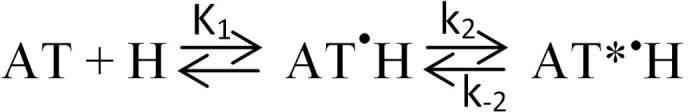
Scheme of binding of antithrombin and heparin. Initial rapid equilibrium, **K**_**1**_, between antithrombin, **AT**, and pentasaccharide, **H**, leads to complex, **AT.H**, followed by rapid conformational change via **k**_**2**_ to a high heparin affinity, highly fluorescence complex, AT*.H.

There are errors in the Van't Hoff equation under the Materials and Methods subsection titled “Determination of denaturing temperature.” Please view the complete, correct equation here:

$$dG=-RTLn\left(Fobs-Fn_{0}\right)/\left(Fd_{0}-Fobs\right)=dHm\left(1-T/Tm\right)$$
