# Supplementary figures and images for: Increased N-Glycosylation Efficiency by Generation of an Aromatic Sequon on N135 of Antithrombin
Source: PLoS One. 2014 Dec 8;9(12):e114454. doi: 10.1371/journal.pone.0114454 (PMC4259341; doi:10.1371/journal.pone.0114454)

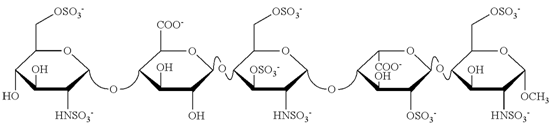

Supplement: S1 Figure — Structure of pentasaccharide. The structure of the essential pentasaccharide sequence present in heparin that is able to activate antithrombin [42]. (TIF) [file pone.0114454.s001.tif]

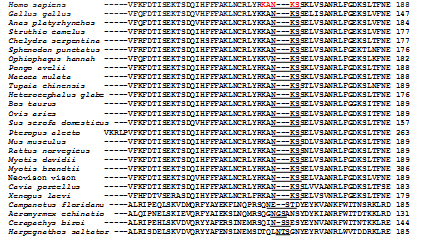

Supplement: S2 Figure — Aligned sequences of 26 antithrombins of different species. These sequences are available at Uniprot (www.uniprot.org/uniprot/). Alignments were performed by clustalW2 (www.ebi.ac.uk/Tools/msa/clustalw2/). The N-glycosylation sequence is underlined and the lysine residue bolded. We excluded fish and other organism sequences because they have not glycosylation consensus sequence on this position. (TIFF) [file pone.0114454.s002.tiff]
