# Supplementary material for: Increased N-Glycosylation Efficiency by Generation of an Aromatic Sequon on N135 of Antithrombin
Source: PLoS One. 2014 Dec 8;9(12):e114454. doi: 10.1371/journal.pone.0114454 (PMC4259341; doi:10.1371/journal.pone.0114454)
Supplement: S1 Table — Primers sequence used for site directed mutagenesis. (PDF) [file pone.0114454.s003.pdf]

Table S1. Primers sequence used for site directed mutagenesis.

Mutations	Nucleotide sequence (5'→3')
**K133F**	GAACTGCCGACTCTATCGATTCGCCAACAAATCCTCCAAG<br>CTTGGAGGATTTGTTGGCGAATCGATAGAGTCGGCAGTTC
**S137T**	CTATCGAAAAGCCAACAAAACCTCCAAGTTAGTATCAGC<br>GCTGATACTAACTTGGAGGTTTTGTTGGCTTTTCGATAG
**A137S**	CTATCGAAAAGCCAACAAATCCTCCAAGTTAGTATCAGC<br>GCTGATACTAACTTGGAGGATTTGTTGGCTTTTCGATAG
**K133F/S137T**	GAACTGCCGACTCTATCGATTTGCCAACAAAACCTCCAAG<br>CTTGGAGGTTTTGTTGGCAAATCGATAGAGTCGGCAGTTC
